# Influence of different aluminum salts on the photocatalytic properties of Al doped TiO_2_ nanoparticles towards the degradation of AO7 dye

**DOI:** 10.1038/s41598-017-08216-2

**Published:** 2017-08-14

**Authors:** Jin-ling Luo, Shi-fa Wang, Wei Liu, Cheng-xiang Tian, Ju-wei Wu, Xiao-tao Zu, Wei-lie Zhou, Xiao-dong Yuan, Xia Xiang

**Affiliations:** 1 School of Physical Electronics, University of Electronic Science and Technology of China, Sichuan Chengdu, 610054 China; 2 Science and Technology on Vacuum Technology and Physics Laboratory, Lanzhou Institute of Physics, Gansu Lanzhou, 730000 China; 3 Advanced Materials Research Institute, University of New Orleans, New Orleans, Louisiana 70148 United States; 4 Research Center of Laser Fusion, China Ac ademy of Engineering Physics, Mianyang, 621900 China

## Abstract

Three kinds of Al-TiO_2_ samples and pure TiO_2_ samples were synthesized via a modified polyacrylamide gel route using different aluminum salts, including Al_2_(SO_4_)_3_∙18H_2_O, AlCl_3_, and Al(NO_3_)_3_∙9H_2_O under identical conditions. The influence of different aluminum salts on the phase purity, morphologies, thermal stability of anatase and photocatalytic properties of the as-prepared Al-TiO_2_ nanoparticles were studied. The energy gap (Eg) of Al-TiO_2_ nanoparticles decreases due to Al ion doping into TiO_2_. The photocatalytic activities of the Al-TiO_2_ samples were investigated by the degradation of acid orange 7 dye in aqueous solution under simulated solar irradiation. The Al-TiO_2_ nanoparticles prepared from Al(NO_3_)_3_∙9H_2_O exhibit the best photocatalytic activity among the four kinds of samples, followed in turn by the Al-TiO_2_ nanoparticles prepared with AlCl_3_, Al_2_(SO_4_)_3_∙18H_2_O and pure TiO_2_. The different performances are attributed to complex effects of Eg, particle size, surface morphology, phase purity and the defect sites of the Al-TiO_2_ nanoparticles.

## Introduction

Photocatalysts have received intense attention as a potential cost-effective approach for addressing various environment and energy-related challenges^1, 2^. The rapid development of nanometer photocatalytic oxidation technology provides a new way to solve the increasingly serious environmental pollution problems such as water, air and soil^3, 4^. Among diverse photocatalysts reported so far, TiO_2_ has attracted growing scientific interest in photocatalytic oxidation of organic molecules due to good chemical stability, absence of toxicity and relative low price^5–8^. In particular, the physical and chemical properties of TiO_2_ in various nanocrystalline forms have been found superior to those bulk counterparts, which are determined by a variety of factors, including their shape, size, crystallinity, phase, composition, etc^9, 10^. Specially, there are three distinct crystallographic forms of titanium dioxide, anatase, brookite, and rutile. Brookite TiO_2_ is hard to synthesize in laboratory. In addition, it is generally accepted that anatase is more active than rutile phase in both photocatalysis and photoelectrochemical studies, which is presumably due to its higher Fermi level than that of rutile by about 0.1 eV^11^. However, the thermodynamic instability of anatase phase is a remarkable characteristic. According to Jaroenworaluck’s literature, rutile appears at temperature of 500–550 °C and becomes the dominant phase at 600 °C^12^. Moreover, Varghese reported that the transformation from anatase to rutile was observed at 430 °C^13^. It is significant to improve thermal stability of anatase in order to enhance the photocatalytic activity, because a higher level of crystallinity can be obtained by annealing titania at high temperatures without forming the rutile phase, which is responsible for the low photocatalytic activity of TiO_2_^14^. In addition, TiO_2_ has some disadvantages such as large band gap and high recombination rate of charge carriers^14^. Therefore, the preparation of high photocatalytic activity of TiO_2_ nanoparticles with these techniques remains a great challenge.

Recently, doping of various metal ions^15^, including 3d transition metal ions^16, 17^ lanthanides^18, 19^, and noble metals^20, 21^, can retard recombination of photo-generated electron-hole pairs in TiO_2_, and improve the photocatalytic activity by extending charge carrier lifetime and visible light response. Miscellaneous mechanisms have been suggested to account for the improved activity of metal ion doped TiO_2_, for instance bandgap narrowing, formation of impurity based energy levels within the bandgap and formation of intrinsic defects such as oxygen vacancies and interstitial Ti^22, 23^. Inspired from the thermal, chemical and mechanical stability and excellent thermal expansion performance of Al-TiO_2_ nano-composite materials, doping of aluminum into TiO_2_ has also been investigated by some researchers^24, 25^. Al-TiO_2_ structures are used in various applications including catalysis, solar cells^26^, and self-cleaning^27, 28^. Zhang *et al*. reported the sol-gel method preparation of a 0.5Al-3%In-TiO_2_ photocatalyst with the maximal decoloration efficiency of 84.3%^29^. Carl Anderson and his coworkers reported the maximal normalized rate constant of photodecomposition of phenol for TiO_2_/Al_2_O_3_ materials was 3.9 × 10^−4^ min^−1^ and the maximal rate constant of photocatalytic decomposition of salicylic acid was less than 1.5 × 10^−2^ min^−1^ ^30^. Hahn *et al*. studied *in situ* Al doped TiO_2_-nanotubes and the corresponding rate constant was 2.5 × 10^−3^ min^−1^ ^31^. However, for nano-sized Al doped TiO_2_, there are potential drawbacks such as severe aggregation and formation of large particles of less activity. In order to achieve the purpose of improving the catalytic performance, some documents have been reported to co-doping of aluminum or other metal ions. Therefore, a new synthesis route is expected to prepare highly dispersed Al doped TiO_2_ nanoparticles and enhance their photocatalytic activity.

Numerous studies describe the approaches to obtain nano-sized Al-TiO_2_ samples^32^, such as chemical vapor synthesis^33^, sol-gel method^34–37^, ball-milling method^38^, wet chemical^39^, citrate gel auto-combustion method^40^ and hydrothermal methods^41–43^. Among them, the sol-gel route is very attractive, offering many advantages like simplicity in the process, high chemical homogeneity at atomic scale, easy control of composition and structure, and ability to tailor particle size and morphology^44^. In the conventional sol–gel process, the gel is built up by chemical and physical bonds between the chemical species and generally a long time is required to achieve the gelation^44^. However, the anatase Al-TiO_2_ nanoparticles, prepared by conventional sol-gel method, have rarely yet been reported to be stable at high temperature. Recently, the polyacrylamide gel has been proved to be an effective and economical facility for easy synthesis of ultrafine oxide powders^5^. Appropriate selection of a chelating agent, monomer systems, initiator, pH value, metal source and sintering temperature can significantly improve the quality of the prepared nanoparticles^45, 46^.

But, most previously reported studies have not investigated the effect of different aluminum salts on the morphology, structure, and photocatalytic activity of the obtained Al-TiO_2_.

In this work, a polyacrylamide gel route is used to synthetize the Al doped TiO_2_ nanoparticles. The influence of different Al sources and annealing temperatures on phase purity, grain size, surface morphology and photocatalytic activity has been investigated. For comparison, pure TiO_2_ was prepared by using the same synthetic method as the Al doped TiO_2_ nanoparticles. The photocatalytic mechanisms of the prepared Al doped TiO_2_ nanoparticles are discussed.

## Results

### Structure and Morphology

**Figure 1:**
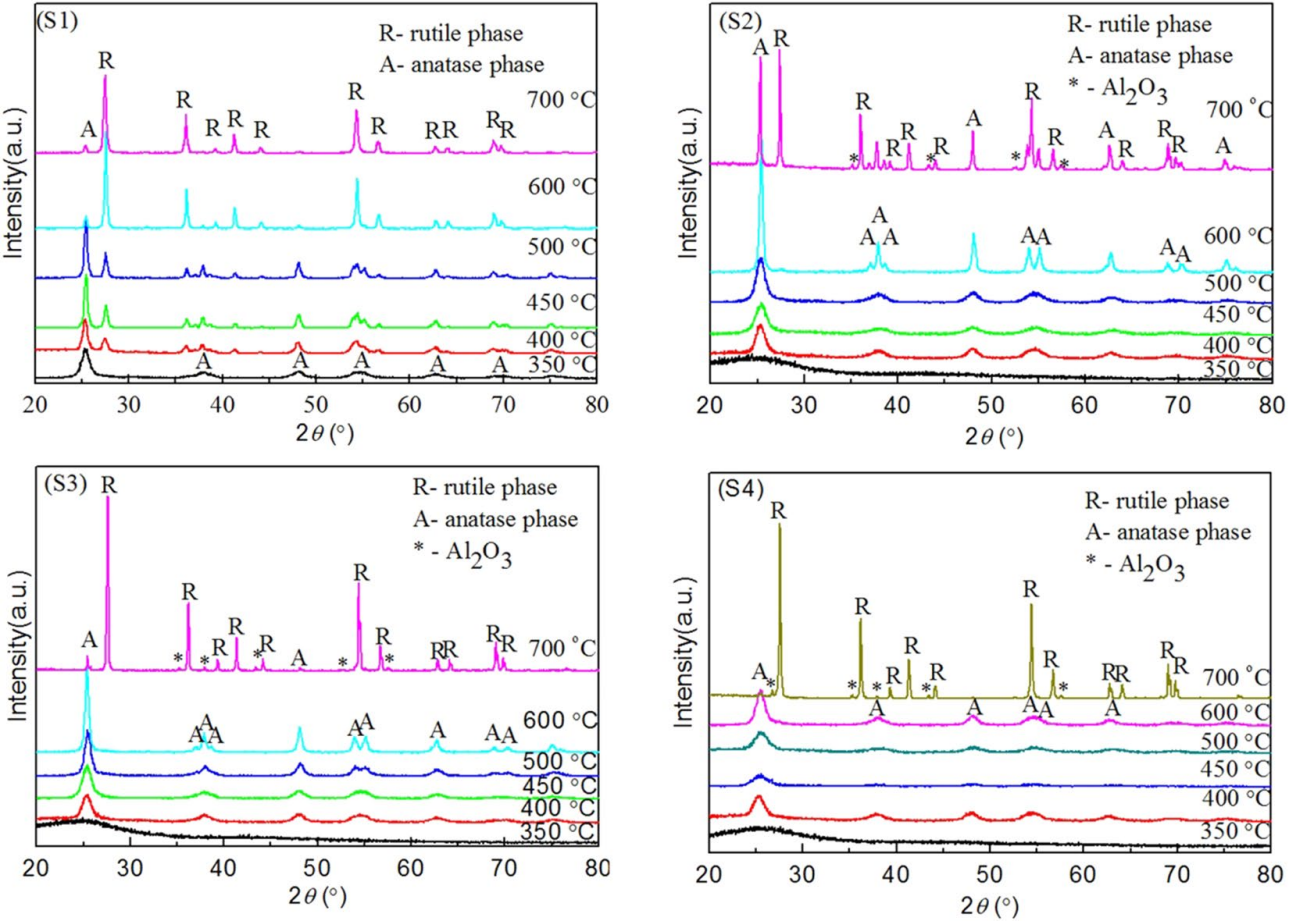
X-ray diffraction patterns of samples S1, S2, S3 and S4 prepared at different calcination temperatures.

Wide-angle X-ray diffraction was used to determine the crystal phase structure of the synthesized titania-based materials (shown in Fig. 1). The observed diffraction peaks at 2θ of 25.44°, 37.14°, 37.96°, 38.74°, 47.94°, 53.72°, 54.84°, 62.40°, 68.38° and 74.50° are attributed to anatase, diffraction peaks at 2θ of 27.58°, 36.18°, 41.30°, 44.14°, 54.12°, 56.38° and 69.80° are attributed to rutile. The XRD results show that none of peaks related to alumina crystal phase is observed in sample S2, S3, S4 until the calcination temperature reaches 700 °C (JCPDS file no. 10–0173), which is ascribed to the high temperature (>700 °C) necessary for alumina crystallization^28^. The average Al-TiO_2_ crystallite size was calculated from the line broadening of corresponding reflections according to Scherrer’s equation^47^

1 $$D=\frac{K\lambda }{\beta \,\cos \,\theta }$$

where *D* is the average crystallite size (nm), *λ* is the wavelength of the X-ray radiation, *k* is a constant taken as 0.9, *β* is the full width at half maximum intensity, and *θ* is the half diffraction angle. The three highest peaks of each XRD pattern were used to calculate the average crystallite size *D* and the results of the average Al-TiO_2_ crystallite size are shown in Table 1. Compared with S1, the addition of Al in sample S2, S3 and S4 lead to smaller crystallite size of Al-TiO_2_. However, when the four xerogel precursor sintered at 700 °C, an abnormal phenomenon was observed. The results could be ascribed to a phase transformation occurred at 700 °C.

**Table 1:** Crystallite size of Al doped TiO_2_ samples.

Sample	350 °C (nm)	400 °C (nm)	450 °C (nm)	500 °C (nm)	600 °C (nm)	700 °C (nm)
S1	8.31826	13.48695	20.26674	20.54383	36.43654	25.91999
S2		6.37416	5.727206	5.660152	22.68439	53.55266
S3		6.623052	5.603304	8.967365	18.11524	61.87919
S4		5.30347	4.735279	5.014893	5.934414	58.40803

As shown in Fig. 1, the sample S1 is the pure anatase TiO_2_ (JCPDS file no. 21–1272) when the calcination temperature is 350 °C but the phase purity of anatase TiO_2_ apparently decreases with the increasing calcination temperature. Structural transformation from anatase to rutile is detected in sample S1 after calciantion at 400 °C (JCPDS file no. 21–1276). However, there is no phase transformation occurred until 700 °C for Sample S2, S3 and S4. The phase content in samples can be calculated by the following equation^48^:

2 $${\rm{Rutile}}\,\,{\rm{phase}}\,\, \% =\frac{100}{1+0.8(\frac{{I}_{A}}{{I}_{R}})}$$

where *I*_*A*_ is the integrated intensity of anatase (101) diffraction peak and *I*_*R*_ is the integrated intensity of rutile (110) diffraction peak^49^. Through calculating, the phase content of rutile in sample S1 is 32.6% when calcined at 400 °C, and it becomes the major phase when the calcination temperature rises to 600 °C. Apparently, the aluminum doping enhanced phase purity of anatase and increased the transformation temperature from anatase to rutile.

The interplanar spacings corresponding to the peaks (Al-TiO_2_ samples sintered at 450 and 600 °C) locating at 2θ = 25.44 (101), 37.96 (004), 48.14 (200), 54.04 (105) and 55.139 (211) are measured and the cell parameters are calculated by the following equation,

3 $$\frac{1}{{d}^{2}}=\frac{{h}^{2}+{k}^{2}}{{a}^{2}}+\frac{{l}^{2}}{{c}^{2}}$$

where *d* is the interplanar spacing of the crystal; *a* and *c* are the lattice parameters; *h*, *k* and *l* are the plane indices. The calculated lattice parameters of the tetragonal Al-TiO_2_ samples are given in Table 2. However, there is a certain degree of deviation of parameters for sample S4 calcined in 450 °C, because few diffraction peaks can be identified from its XRD due to the low crystallinity. In addition, the standard deviations of lattice parameter *a* and *c* are also calculated using the equations (4) and (5).

4 $${\delta }_{a}=\sqrt{\frac{{\sum }_{i-1}^{n}{({a}_{i}-\bar{a})}^{2}}{n}}$$

5 $${\delta }_{c}=\sqrt{\frac{{\sum }_{i-1}^{n}{({c}_{i}-\bar{c})}^{2}}{n}}$$

Where *δ*_*a*_ and *δ*_*c*_ are the standard deviation of lattice parameters *a* and *c*, respectively; $\bar{a}$ and $\bar{c}$ are the mean value of lattice parameters *a* and *c*, respectively. The calculated standard deviation results have been shown in Table 2. The minimum values of standard deviation were ${\delta }_{a}=0.0042$ and ${\delta }_{c}=0.0126$ for the sample S3 sintered at 600 °C. It indicates that the calculated lattice parameter is credible. The unit cell volume of tetragonal Al-TiO_2_ was calculated by the following equation

6 $${\rm{V}}={a}^{2}c$$

**Table 2:** Unit cell volumes, cell parameters and standard deviation of cell parameters of main peaks of Al-TiO_2_ samples S1, S2, S3 and S4 calcined at 450 and 600 °C.

Sample	unit cell volume (Å^3^)	d of Al-TiO_2_ diffraction peaks/(Å)				Cell parameters			
		(101)	(004)	(200)	(211)	a(Å)	δa	c(Å)	δc
S1–450	136.24	3.4983	2.3706	1.8961	1.6728	3.7621	0.0072	9.4669	0.0623
S1–600	135.13	3.4956	2.3710	1.8873	1.6622	3.7646	0.0437	9.4933	0.5982
S2–450	135.95	3.4904	2.3688	1.8939	1.6704	3.7749	0.0222	9.2404	0.3323
S2–600	135.18	3.4983	2.3683	1.8886	1.6643	3.7637	0.0078	9.5259	0.0689
S3–450	134.90	3.5037	2.3710	1.8856	1.6800	3.7736	0.0323	9.4819	0.4991
S3–600	135.48	3.5037	2.3719	1.8894	1.6627	3.7704	0.0042	9.5110	0.0126
S4–450	180.54	3.4808	2.3757	1.8889	1.6651	3.7778	0.0072	9.5028	0.3658
S4–600	133.54	3.4769	2.3505	1.8843	1.6568	3.7540	0.0150	9.3281	0.1897

The calculated unit cell volumes of these samples are shown in Table 2 as well. It indicates that the effect of Al on enhancing thermal stability and controlling crystal growth of anatase TiO_2_ cannot be neglected. The improved thermal stability is useful to improve the photocatalytic activity, because the higher level of TiO_2_ crystallinity can be obtained by annealing at higher temperatures without forming the rutile structure of low photocatalytic activity^14^.

**Figure 2:**
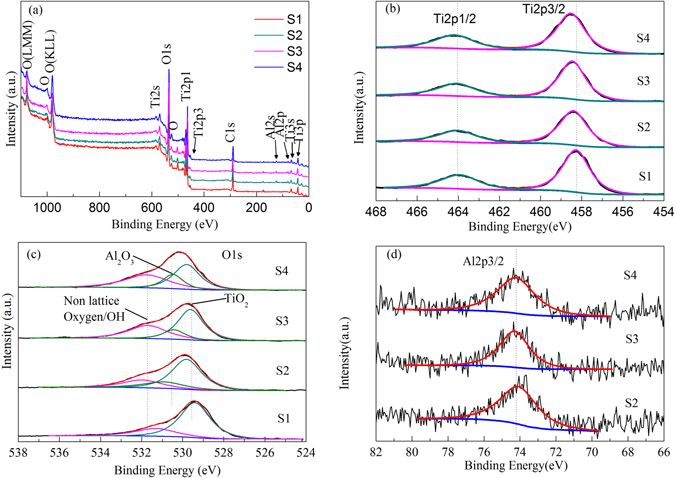
XPS obtained for: (**a**) wide XPS surveys of Samples S1, S2, S3 and S4 calcined at 450 °C, (**b**) Ti_2p_ peak, (**c**) O_1s_ peak, (**d**) Al_2p_ peak in the four samples calcined at 450 °C.

To determine the bonding state of the Al doped TiO_2_ nanoparticles, XPS characterization was performed to explore the Ti_2p_, O_1s_, and Al_2p_ spectra of sample S1, S2, S3 and S4 calcined at 450 °C, as shown in Fig. 2. Figure 2(a) shows wide XPS surveys of the S1, S2, S3, and S4 samples calcinated at 450 °C. The electron binding energies of Ti_2p3/2_ and Ti_2p1/2_ in pure TiO_2_ sample (S1) are 458.27 and 463.93 eV, respectively, as shown in Fig. 2(b). After doping of aluminum into materials, the binding energies of Ti_2p3/2_ and Ti_2p1/2_ in Al doped TiO_2_ samples increase in the range of 0.09–0.24 eV, indicating the binding energy of Ti_2p_ electron shifting to higher energy. The Pauling electronegativity of aluminum ion is larger than that of titanium ion, which will induce electron transfer from Ti to Al, thus resulting in an increase in binding energy of Ti_2p_ electron^50^.

The O1s XPS spectra of pure TiO_2_ and Al-TiO_2_ are shown in Fig. 2(c). The O1s XPS spectrums of Al-TiO_2_ are fitted with three peaks at ~529.7 eV, ~530.6 eV and ~531.7 eV, which are attributed to Ti-O, Al-O and non-lattice oxygen. In the O1s XPS spectrums of pure TiO_2_, only two peaks at binding energies 529.5 eV and 531.3 eV are observed which are attributed to Ti-O and surface absorbed OH group^51^.

The Al phase in the prepared samples maybe existed as amorphous Al_2_O_3_ through the analysis of the O1s core level spectra. Al_2_O_3_ xerogel prepared by the same method shows a γ- Al_2_O_3_ phase when sintered at 700 °C^52^, however, in this case, the Al-TiO_2_ xerogel only sintered at 450 °C and the γ- Al_2_O_3_ phase is unobserved as shown by XRD patterns (See Fig. 1). Amorphous Al_2_O_3_ should have a high electron-transfer ability from TiO_2_ because amorphous materials should contain more defect sites than crystals, and Al_2_O_3_ provides more adsorption sites in the vicinity of TiO_2_.

As can be seen from Fig. 2(c), oxygen from titania lattice is also the dominant source of oxygen. Meanwhile, the hydroxyl species slightly increases after doping of aluminum ions in the materials. The existence of hydroxyl species is beneficial to photocatalytic activity since hydroxyl radicals can easily form through oxidizing when the surface hydroxyl species are available^50^. The amount of amorphous Al_2_O_3_ in Al-TiO_2_ samples is differen with the differen aluminum salts doping in the preparation procedure, thus providing different amount of adsorption sites in the vicinity of TiO_2_. Figure 2(d) shows XPS spectra of Al_2p3/2_ for doped samples. The peaks near 74.27 eV and the intensity of three peaks for S2, S3 and S4 are similar because of the same dopant concentration. The real content of Al in the samples have been detected by XPS. The results have been shown in Table 3. The results indicate that the order of Al content for samples S2, S3 and S4 should be S2 > S3 > S4.

**Table 3:** XPS parameters of four kinds of samples sintered at 450 °C.

at%	S1	S2	S3	S4
C	33.65%	23.45%	25.05%	32.20%
O	50.55%	57.62%	56.95%	50.29%
Ti	15.79%	15.63%	14.75%	14.47%
Al		3.30%	3.26%	3.04%

**Figure 3:**
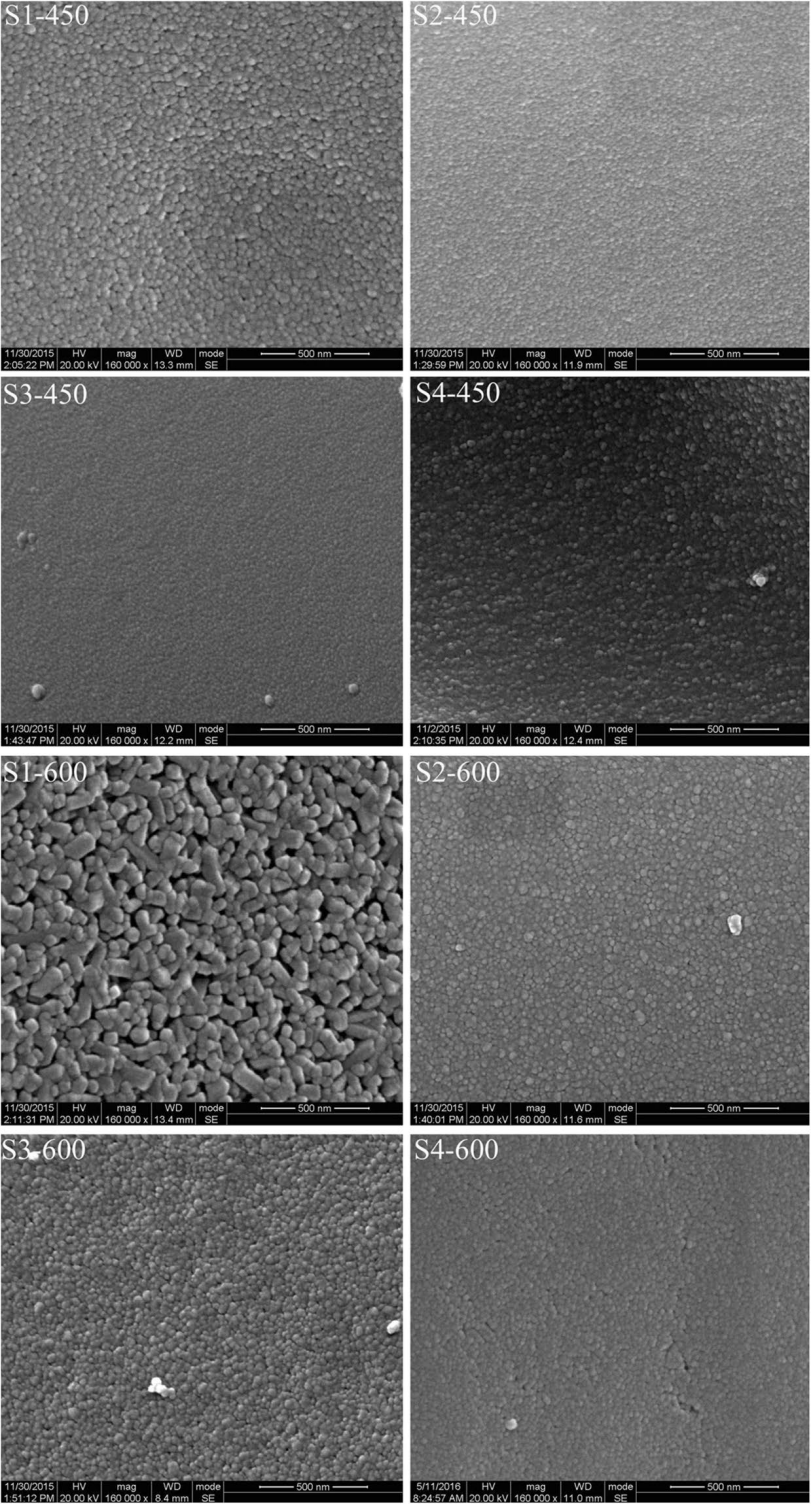
SEM images of Samples S1, S2, S3 and S4 calcined at 450 and 600 °C.

Figure 3 shows the SEM images of S1, S2, S3, and S4 samples calcined at 450 °C and 600 °C. The Al-doped particles fabricated from different aluminum salts are approximately spherical and well-dispersed, and there is no aggregation phenomenon. The average particle size increases with the increasing calcination temperature. At the same sintering temperature, it’s clear that the particle sizes of S2, S3 and S4 are all smaller than that of S1. When the calcination temperature is 600 °C, there are obvious adhesion and agglomerates in sample S1. While for sample S2, S3 and S4, the average particles sizes are also small and there is no aggregation and adhesion even after calcination at 600 °C. The SEM images demonstrate the effect of Al on the modification of crystal morphology of TiO_2_ samples. Doping of Al within the TiO_2_ matrix inhibits the grain growth and agglomeration of TiO_2_ crystals during synthesis process.

**Figure 4:**
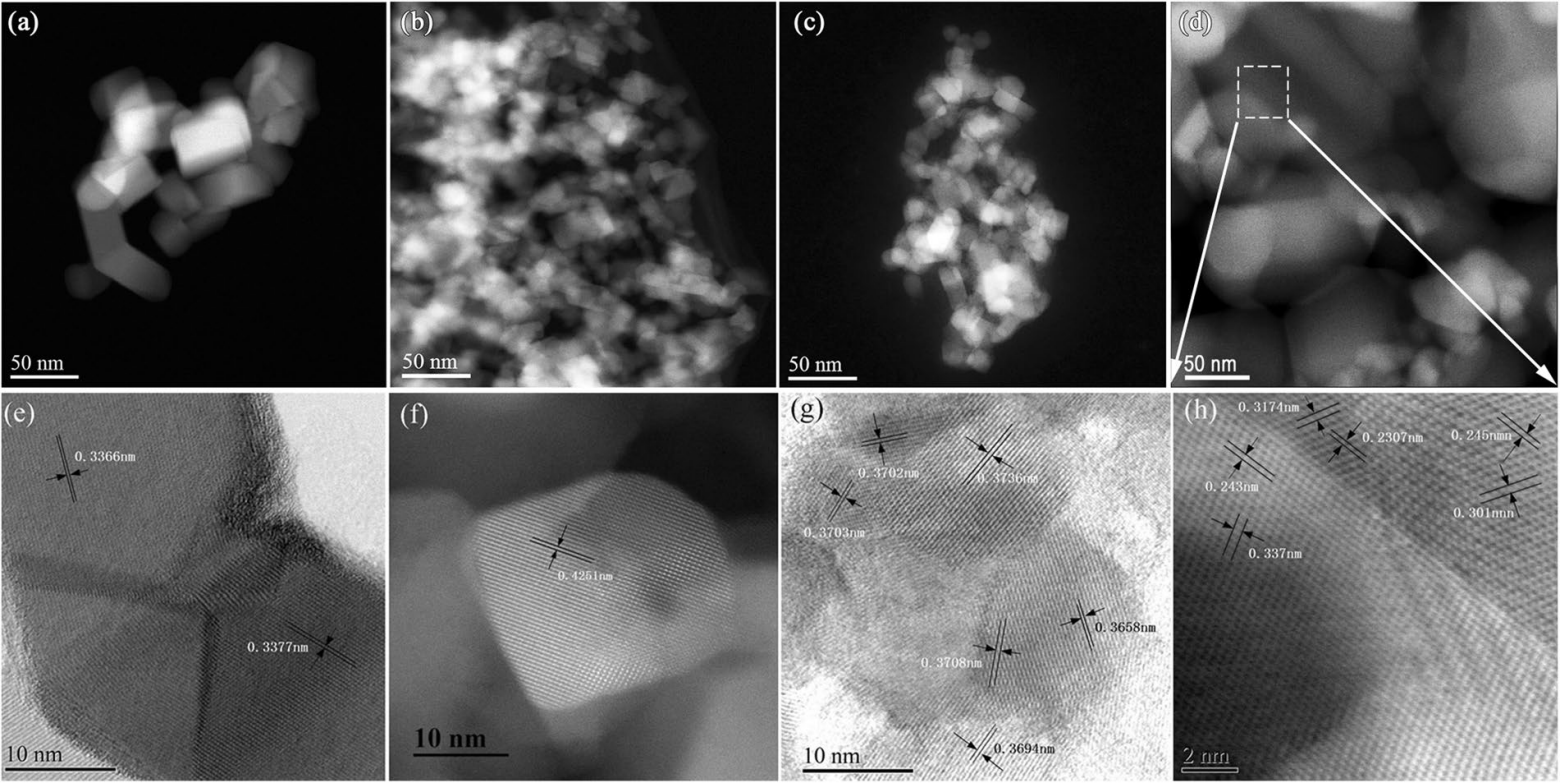
TEM micrographs of TiO_2_ nanoparticles after calcining at 600 °C for 5 h doped with different Al salts. (**a**,**e**) S1, (**b**,**f**) S2, (**c**,**g**) S3, (**d**,**h**) S4.

TEM images for TiO_2_ nanoparticles synthesized with different alumina salts calcined at 600 °C are shown in Fig. 4. The average particle size is ~25 nm in diameter, as shown in (a), (b), (c) and (d) in Fig. 4. The TiO_2_ nanocrystals are highly crystallized, as demonstrated from the well-resolved lattice features in the high-resolution TEM (HRTEM) image (Fig. 4 (e,f,g and h)). Although the particle size of S4 is the biggest among four samples as shown in Fig. 4, but the S4 particles are formed by twin grains and the other samples are made up of single crystals, such as particle size of the XRD analysis described. If the grain size is compared, the grain size of sample S4 is not even larger than that of other samples, and even smaller, as shown in the XRD patterns. In addition, TEM shows that the crystallinity and dispersibility of S4 are the best.

**Figure 5:**
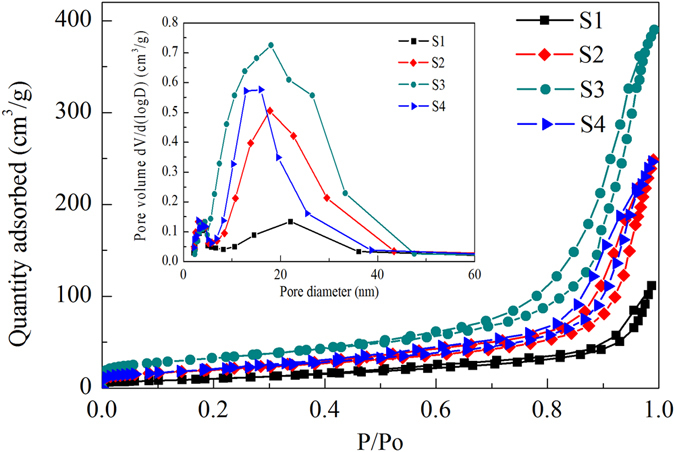
N_2_ adsorption-desorption isotherms of as-prepared samples calcinated at 600 °C, the inset is the pore size distribution acquired using the BJH method from the desorption branch of isotherm.

N_2_ adsorption-desorption isotherms of as-prepared samples calcinated at 600 °C are plotted in Fig. 5, and the pore size distribution acquired using the BJH method from the desorption branch of isotherm is shown as the inset. The isotherms are type-IV isotherm with an H_3_ type hysteresis loop according to the IUPAC classification, indicating that samples possess mesopores. The specific surface area have been shown in Table 4. It can be seen that the specific surface area of Al-TiO_2_ samples are much larger than that of pure TiO_2_ (S1). The larger surface area in the Al-TiO_2_ samples confirms XRD results, and is able to provide more active sites for photodegradation of dye.

**Table 4:** BET specific surface area, Eg and degradation percentage of Al-TiO_2_ samples S1, S2, S3 and S4 calcined at 450 °C and 600 °C.

Sample	BET specific surface area(m^2^/g)	Eg (eV)		Degradation	
				Percentage (%)	
	600 °C	450 °C	600 °C	450 °C	600 °C
S1	36.11	3.23	3.25	55.05	3.05
S2	69.85	3.11	3.00	61.38	39.01
S3	115.49	3.06	3.03	73.80	62.12
S4	71.98	3.13	3.21	98.87	93.32

### Raman Analysis

**Figure 6:**
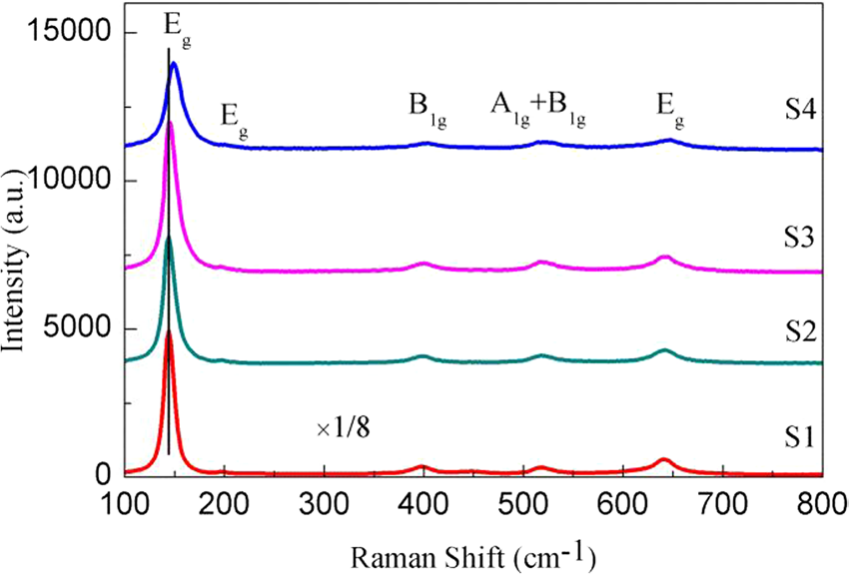
Raman spectrum of Samples S1, S2, S3 and S4 calcined at 450 °C.

In order to further confirm the dominant composition of Al-TiO_2_, Raman analysis was conducted. Figure 6 illustrates the Raman spectra of four samples of TiO_2_ nanoparticle calcinated at 450 °C. According to factor group analysis, anatase has six Raman active modes (A1g + 2B1g + 3Eg). For samples S1, S2, and S3, the appearance of sharp and intense peak at 144.30 cm^−1^ and a very weak peak at around 197.47 cm^−1^ assigned to E_g_ mode of vibration is the characteristic of anatase TiO_2_. In the higher frequency region, the other peaks at around 400, 519 and 641 cm^−1^ corresponding to B_1g_, A_1g_ + B_1g_ and E_g_ modes also confirm the presence of anatase TiO_2_^53, 54^ in samples. It’s worth to be mentioned that the characteristic peak of S4 located at 147.85 cm^−1^ shifts to a 3.55 cm^−1^ higher position than other samples that locate at 144.30 cm^−1^. Compared to the pure TiO_2_ (sample S1), peak intensities of sample S2, S3 and S4 relatively decrease after Al ion doping. They are all due to the decreased crystallite size of Al doped TiO_2_ samples^55, 56^.

### Evaluation of the photocatalytic properties

**Figure 7:**
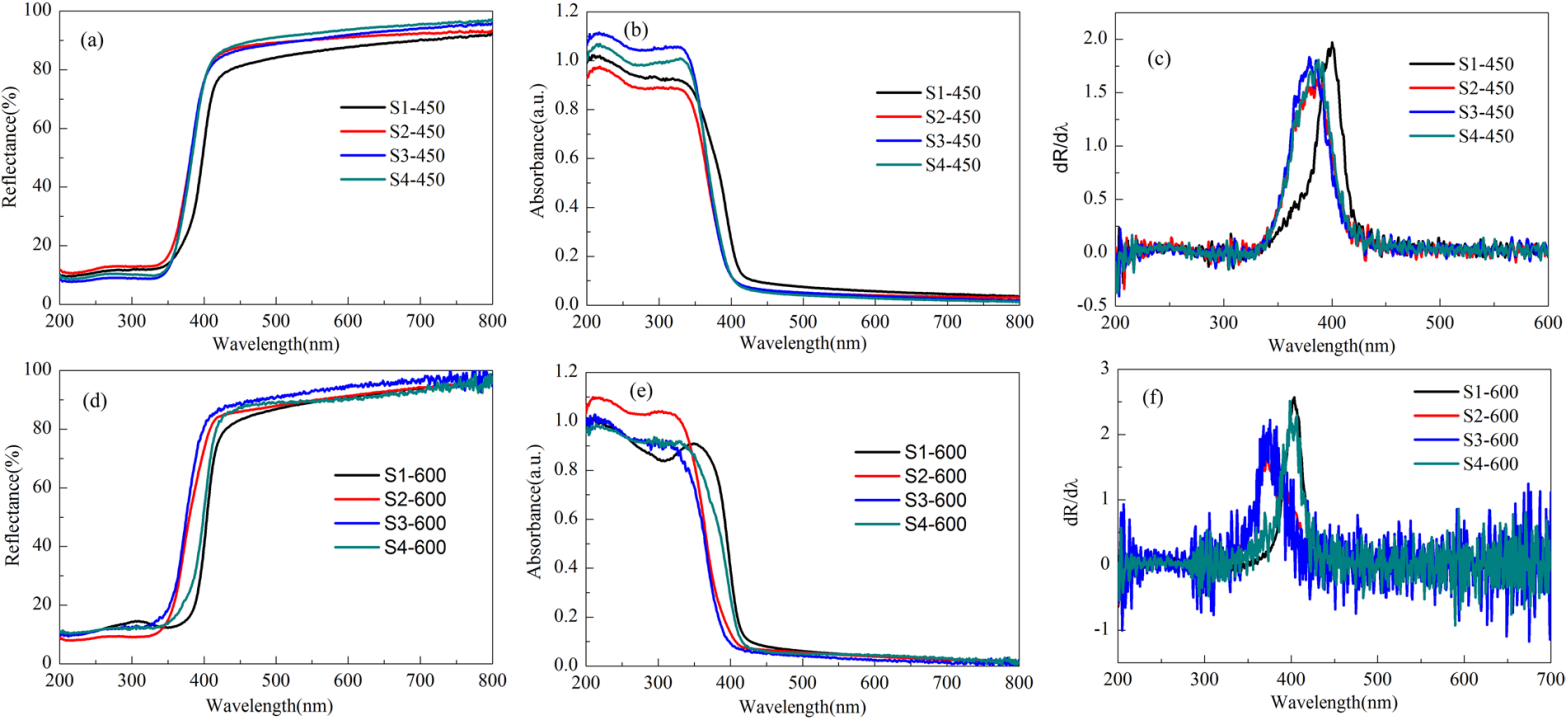
(**a**) UV-visible diffuse reflectance spectra, (**b**) UV-visible absorption spectra and (**c**) Eg values of Samples S1, S2, S3 and S4 calcined at 450 °C. (**d**) UV-visible diffuse reflectance spectra, (**e**) UV-visible absorption spectra and (**f**) Eg values of Samples S1, S2, S3 and S4 calcined at 600 °C.

Figure 7(a,d) show the UV-visible diffuse reflectance spectra of Samples S1, S2, S3 and S4 calcined at 450 and 600 °C, and Fig. 7(b,e) give the UV-visible absorption spectra of Al doped TiO_2_ samples which are transformed from Fig. 7(a,d) respectively according to the Kubelka-Munk (K-M) theory. Simultaneously, the corresponding first derivative of the reflectance (R) with respect to wavelength λ (i.e., dR/dλ) is obtained, as shown in Fig. 7(c,f), where the peak wavelength is characterized to be the absorption edge of the samples, from which the energy gap (Eg) of the samples can be calculated as shown in Table 4.

**Figure 8:**
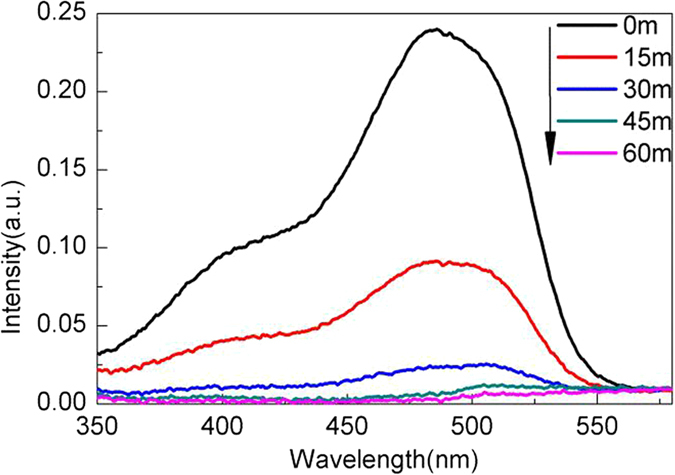
UV-visible absorption spectra of AO7 solution at different irradiation time in presence of sample S4 calcined at 450 °C.

The photocatalytic activity of the Al doped TiO_2_ samples were evaluated by the degradation of AO7 under UV light irradiation. Figure 8 shows some of UV–visible absorption spectra for AO7 solution during illumination in presence of sample S4 calcined at 450 °C. The absorption intensity of AO7 solution decreases apparently with the increasing irradiating time due to the degradation of dye molecules. After 45 min of irradiation, there is only very weak absorption of the solution. And it can be completely degraded within one hour.

**Figure 9:**
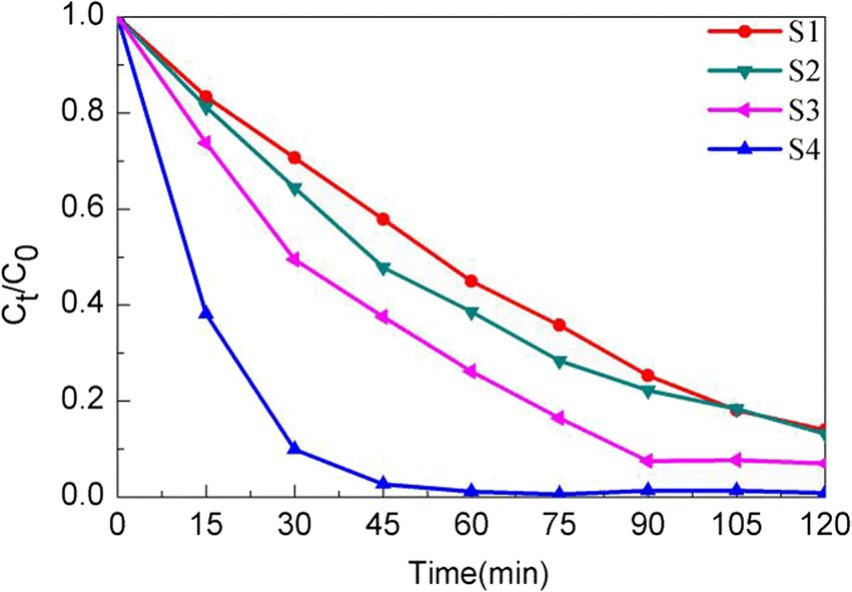
The change in the relative amount of the AO7 during the irradiation using as-prepared samples calcined at 450 °C.

**Figure 10:**
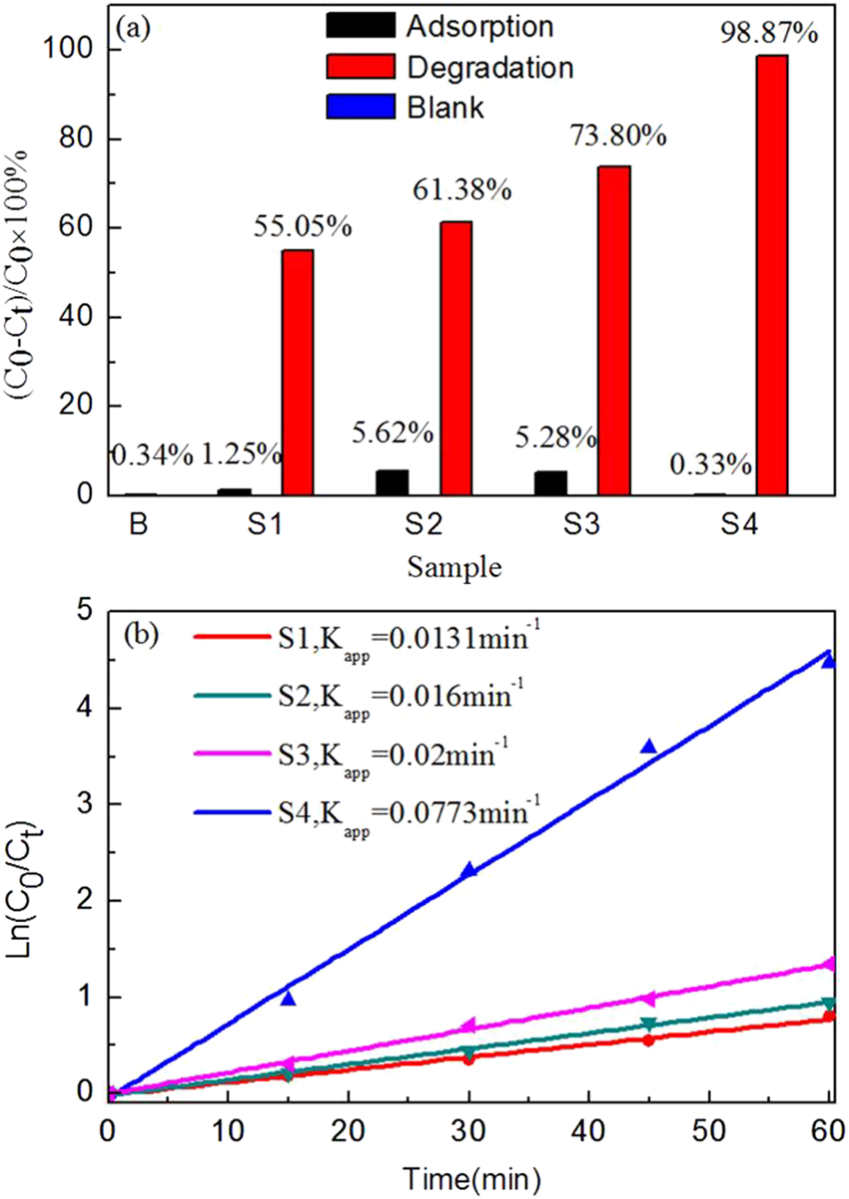
(**a**) Degradation percentage of AO7 after irradiation for 1 h using the Al-TiO_2_ samples calcined at 450 °C, (**b**) Kinetic plots of AO7 degradation in presence of the Al-TiO_2_ samples calcined at 450 °C.

The change of the relative amount of the AO7 solution during irradiation for the samples calcined at 450 °C is shown in Fig. 9, which demonstrates that S4 photolysis brings about a concentration decrease about 99% after 1 h irradiation. However for the sample S1, its concentration still remains higher than 10% after 2 h irradiation. Figure 10(a) shows the degradation percentage of AO7 after irradiation for 1 h along with results of the reference experiment and adsorption experiment, where the initial concentration of AO7 is 5 mg/L and the content of catalyst is 0.5 g/L. There is only AO7 and no catalyst in the solution under irradiation in the reference experiment, while in the adsorption experiment AO7 and catalyst is dissolved in the solution but on irradiation conditions. The degradation percentage is defined as (C_0_ − C_t_)/C_0_ × 100%, where C_0_ and C_t_ are the AO7 concentrations before and after irradiation, respectively. AO7 appears to be stable without photocatalyst under short-time UV irradiation, and its degradation percentage is 0.34% after 1 h UV irradiation. In the absence of UV light, the Al-TiO_2_ samples exhibit the adsorption toward AO7 that are less than 6%. However, for irradiation with UV light in the presence of Al-TiO_2_ samples, the degradation of the AO7 is markedly enhanced, implying that the samples exhibit a good photocatalytic activity.

The highest photocatalytic activity is observed for sample S4, where the degradation percentage of AO7 is about 98.87% after irradiation for 1 h. The sample S1 exhibits a relatively inferior photocatalytic activity among those samples, which is probably due to its poor morphology, large-size particles and the substantial presence of rutile in the sample. Generally, small particle size and large surface area to volume ratio is required to achieve good photocatalytic activity since the photocatalytic reaction occurs dominantly on the catalyst surface^44^. Figure 10(b) shows the plots of ln(C_0_/C_t_) versus irradiation time (t). The photodegradation of AO7 on the Al-TiO_2_ samples can be well modeled using the first-order kinetic equation, ln(C_0_/C_t_) = k_app_t, where k_app_ is the apparent first-order reaction rate constant (min^−1^). In each case, the correlation coefficient R^2^ is not less than 0.99. It is seen that the first-order reaction rate constant k_app_ for the four samples given in Fig. 10(b) follows the sequence: S4 > S3 > S2 > S1. According to the BET specific surface area of four samples, the increase of BET specific surface area has led to the increase of the photocatalytic activity, except for the sample S3. The results indicate that the photocatalytic activity is relevant to not only the BET specific surface area, Eg and active sites but also the hydroxyl radical of Al-TiO_2_ samples.

**Figure 11:**
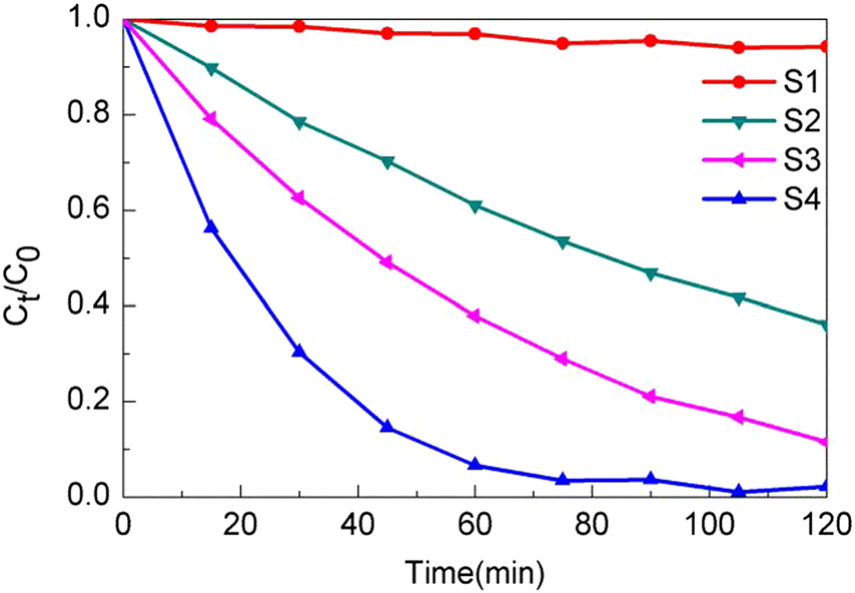
The change in the relative amount of the AO7 during the irradiation using as-prepared samples calcined at 600 °C.

**Figure 12:**
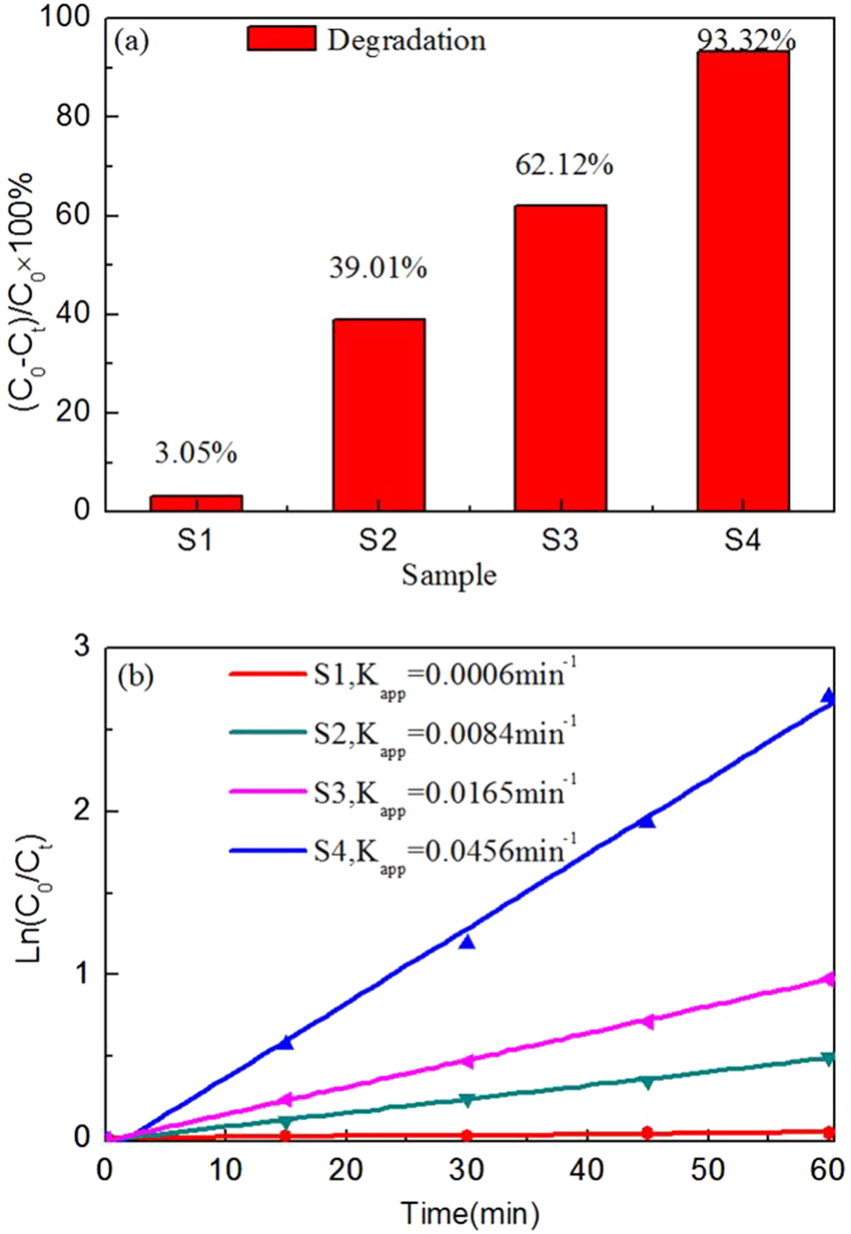
(**a**) Degradation percentage of AO7 after irradiation for 1 h using the Al-TiO_2_ samples calcined at 600 °C, (**b**) Kinetic plots of AO7 degradation in presence of the Al-TiO_2_ samples calcined at 600 °C.

Similar to Figs 9, 10 and 11 displays the change of the relative amount of the AO7 solution during irradiation for the samples calcined at 600 °C and Fig. 12 shows the degradation percentage of AO7 after irradiation for 1 h and the plots of ln(C_0_/C_t_) versus irradiation time (t). It demonstrates that S4 photolysis almost photodegrade the AO7 completely after 75 min irradiation, however after 2 h irradiation, the photodegradation of AO7 in presence of S1 photolysis exhibits almost no effect as shown in Fig. 11. The degradation effect also follows the sequence: S4 > S3 > S2 > S1. As shown in Fig. 12(a), the highest photocatalytic activity is also observed for sample S4, where the degradation percentage of AO7 is about 93.32% after 1 h irradiation. Accordingly, 62.12%, 39.01%, and 3.05% are corresponding to S3, S2, and S1 separately. The sample S1 exhibits worst photocatalytic effect among those samples. In each case, the correlation coefficient R^2^ is not less than 0.99. It is seen that the first-order reaction rate constant k_app_ for the four samples given in Fig. 12(b) follows the same sequence: S4 > S3 > S2 > S1.

With the calcination temperature changing from 400 °C to 600 °C, there is a certain degree of decrease of the catalytic efficiency of four samples, especially for sample S1. It maybe because rutile has become the main crystalline phase of S1 calcined at 600 °C and its crystal morphology is very poor, appearing serious agglomeration. However, S2, S3, S4 are still anatase and the grain sizes of them don’t increase too much.

**Figure 13:**
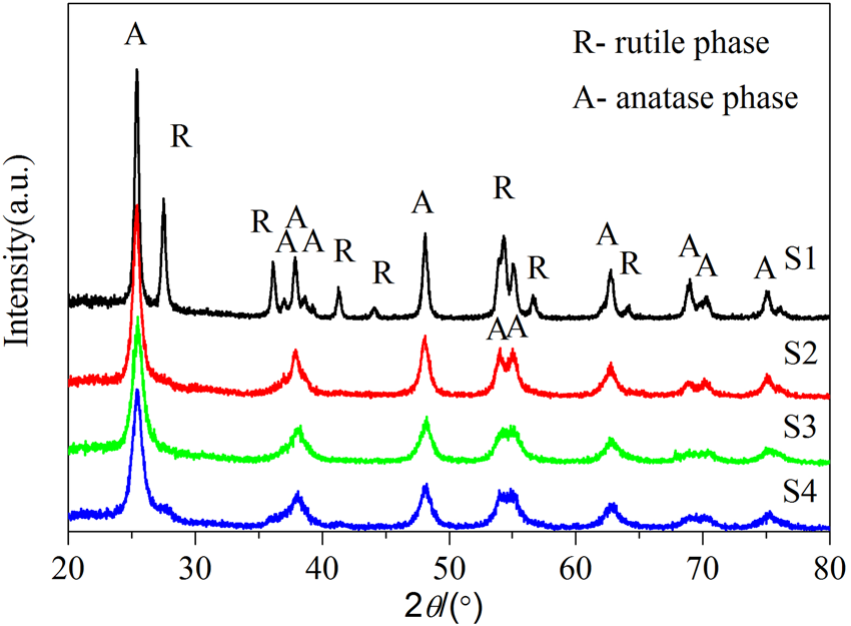
X-ray diffraction patterns of four kinds of samples calcined at 450 °C after the photocatalytic experiments.

In order to promote its technological applications in photocatalysis, the catalyst is required to be well stable in its structure. Figure 13 shows the XRD patterns of Al-TiO_2_ nannoparticles after the photocatalytic experiments, which samples are calcined at 450 °C. It can be seen that the XRD of four kinds of samples measured after the photocatalytic experiment is almost the same as that measured before the photocatalytic experiment shown in Fig. 1. This demonstrates that as-prepared samples undergo no structural changes during the photocatalytic experiments, which exhibit a good photostability.

## Discussion

Some studies were performed to explain the role of the aluminum and its compounds versus the titania photocatalytic activity^57^. It is generally presumed that the active species responsible for the photocatalytic degradation of pollutants are the hydroxyl radicals (·OH) due to their high oxidation potential^58^. A photoexcited electron is promoted from the valence band (VB) to the conduction band (CB) when catalyst is irradiated with light that is higher than its E_g_. This reaction leaves a positive hole in the valence band and a negative electron in the conduction band, thus creating an electron–hole pair (e^−^h^+^). The positive hole in the valence band can oxidize the OH^−^ or water at surface to produce hydroxyl radical (·OH) which acts as extremely powerful oxidant of organic pollutants. The photo-excited electron located in the conduction band is reduced to form the superoxide radical anion (O_2_·) upon reaction with oxygen and hydroperoxide radical (·OOH) upon further reaction with H^+ ^^59^. Hydroxyl radical (·OH) have been proved to be the main active species generated in the photocatalytic process responsible for the degradation of pollutants in many articles^55, 60^.

**Figure 14:**
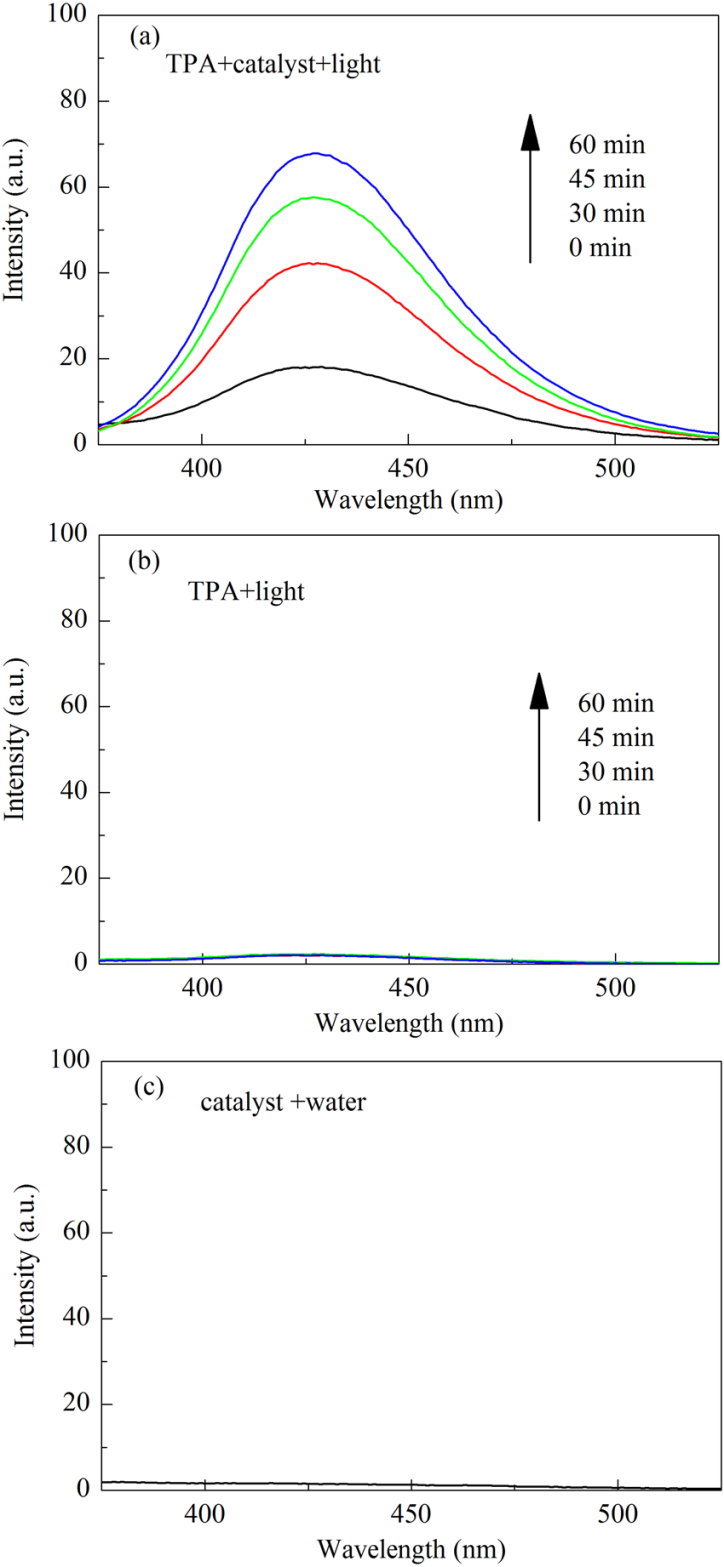
PL spectra of (**a**) the TPA solution with catalyst irradiated for different time, (**b**) the TPA solution without catalyst irradiated for different time, (**c**) aqueous solution dissolved with unirradiated catalyst.

Figure 14(a) shows the PL spectra of TPA solution reacted for different times over the irradiated Al-TiO_2_ nanoparticles. It is seen that the TPA reaction solution shows PL signal centered around 429 nm, and its intensity increases with increasing irradiated time. The indicates that ·OH are produced over the irradiated Al-TiO_2_ nanoparticles. Figure 14(b,c) show the PL spectra of TPA solution without catalyst irradiated for different times and aqueous solution dissolved with unirradiated catalyst. They all show a very weak PL signal, indicating the production of the vast majority of ·OH are from the irradiated catalyst and ·OH are suggested to be the dominant active species responsible for the dye degradation.

**Figure 15:**
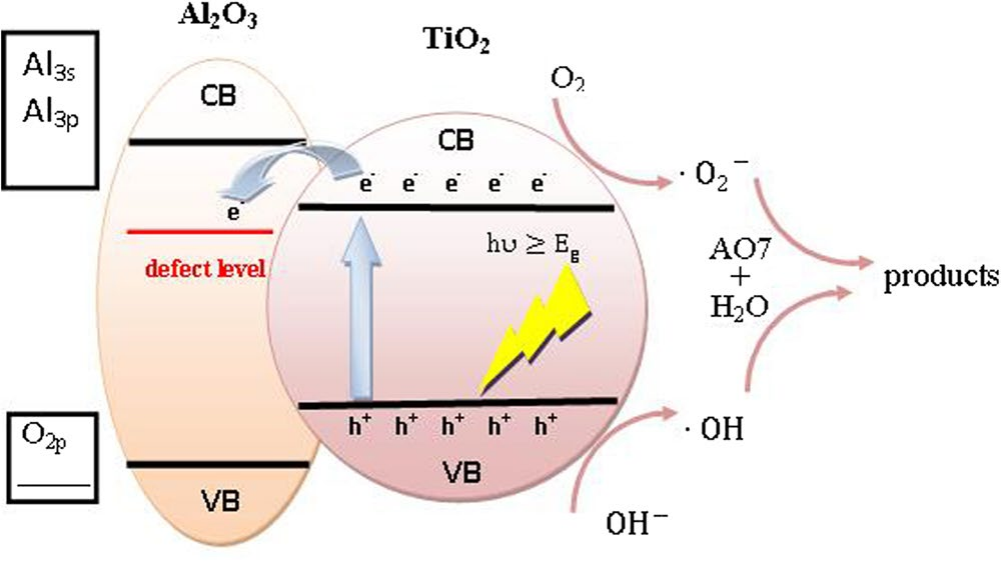
Schematic illustration of the photocatalytic process.

The possible degradation mechanism of AO7 over Al doped TiO_2_ material under light irradiation is shown in Fig. 15. The TiO_2_ behaves as the photoactive center, e.g., generating hydroxyl radicals under irradiation. While amorphous Al_2_O_3_ should have a high electron-transfer ability from TiO_2_ because amorphous materials should contain more defect sites than crystals. Thus, the transference of photoinduced electrons to the defect levels of amorphous Al_2_O_3_ benefits the separation of electrons and holes, which enhances the quantum yield of TiO_2_^39^. Furthermore, the Al_2_O_3_ provides more adsorption sites in the vicinity of TiO_2_^30^ and decrease the grain size of anatase, thus increasing the photoactive surface area of the prepared materials^61^.

In summary, Al-TiO_2_ nanoparticles with different aluminum salt doping have been synthesized by a modified polyacryamide gel route. The as-prepared particles exhibit that different aluminum salts have nonnegligible effect on phase purity, structure, grain size, surface morphology and photocatalytic properties. The sizes of nanoparticles in pure TiO_2_ sample increase and obvious adhesion and agglomeration occur with the increase in calcination temperature. However, the addition of Al in samples S2, S3 and S4 hinders grain growth and agglomeration, thus leading to crystallite size of anatase shrinking, enhancing the phase purity and increasing the transformation temperature of anatase to rutile, which are all beneficial to photocatalytic activity. The investigation of the UV-visible diffuse reflectance spectra reveals that the energy gap (Eg) of Al-TiO_2_ samples narrow after aluminum doping. The photocatalytic tests show that the Al-TiO_2_ nanoparticles exhibitit superior photocatalytic activity for the decomposition of AO7, especially for sample S4 which takes Al(NO_3_)_3_·9H_2_O as aluminum salt.

## Methods

### Preparation of Al doped TiO_2_ nanoparticles

**Figure 16:**
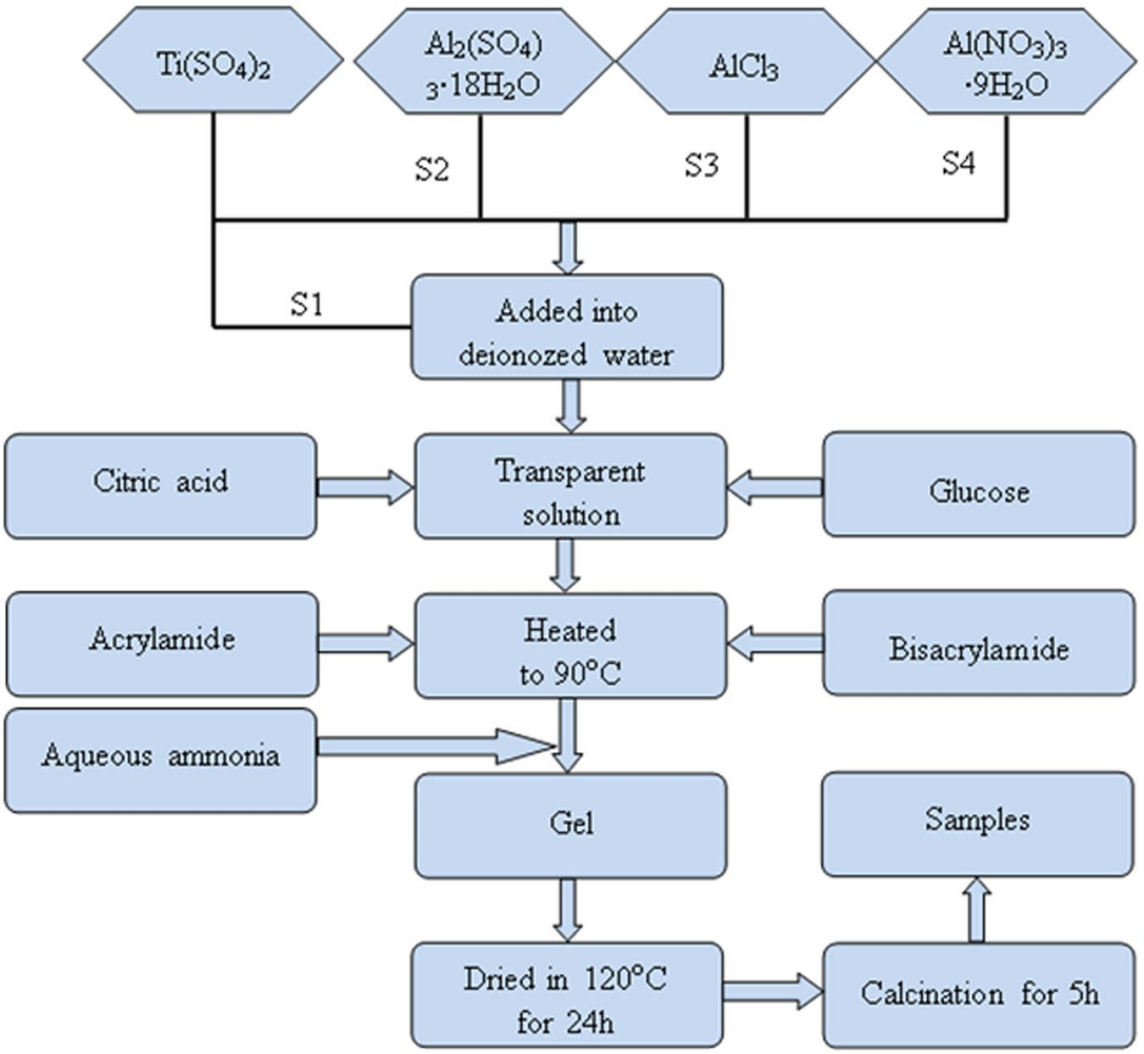
Flowchart of Al-TiO_2_ nanoparticles synthesized via a modified polyacrylamide gel route.

In this study, the Al doped TiO_2_ samples were prepared with three different aluminum salts: S1-pure TiO_2_ without Al doped, S2-Al_2_(SO_4_)_3_·18H_2_O, S3-AlCl_3_, S4-Al(NO_3_)_3_·9H_2_O. The raw materials of experiments for samples S1, S2, S3 and S4 are given in Table 5. Taking the Al(NO_3_)_3_·9H_2_O for example, a certain stoichiometric amount of Ti(SO_4_)_2_ and Al(NO_3_)_3_·9H_2_O were dissolved in the deionized water to obtain a solution of 0.015 mol/L that the molar ratio of Ti:Al is 9:1. After the solution became transparent, a stoichiometric amount of chelating agent (citric acid) was added to the solution in the molar ratio 1.5:1 with respect to the cations (Ti, Al) to complex the cations. After that, 20 g glucose was dissolved in the solution. Finally, the acrylamide and N,N’-methylene-bisacrylamide monomers were added to the solution. The total amount of the used monomers in each case is same and was 9 times amount (in mole) of the cations. The resultant solution was heated to 90 °C on a hot plate to initiate the polymerization reaction, and a few minutes later a polyacrylamide gel was formed. The gel was dried at 120 °C for 24 h in a thermostat drier. The obtained xerogel precursor was ground into powder and some powder was sintered at 350, 400, 450, 500, 600 and 700 °C for 5 h to prepare Al doped TiO_2_ samples. The flowchart for preparation of Al doped TiO_2_ sample via the modified polyacrylamide gel route is shown schematically in Fig. 16 ^46^.

**Table 5:** The raw materials of Al doped TiO_2_ samples.

Sample	Al_2_(SO_4_)_3_∙18H_2_O	AlCl_3_	Al(NO_3_)_3_∙9H_2_O	Ti(SO_4_)_2_	Citric acid	glucose	acrylamide	Bis-acrylamide
S1	/	/	/	3.2399 g (0.0135 mol)	4.7282 g	20 g	9.5958 g	1.9192 g
S2	0.4998 g (0.00075 mol)	/	/	3.2399 g (0.0135 mol)	4.7282 g	20 g	9.5958 g	1.9192 g
S3	/	0.1999 g (0.0015 mol)	/	3.2399 g (0.0135 mol)	4.7282 g	20 g	9.5958 g	1.9192 g
S4	/	/	0.5627 g (0.0015 mol)	3.2399 g (0.0135 mol)	4.7282 g	20 g	9.5958 g	1.9192 g

### Sample Characterization

The crystalline phases of the samples were identified using X-ray diffractometer (XRD, Rigaku D/max-2500) with Cu Kα radiation at a wavelength of 1.5406 Å operated at 40 kV and 30 mA. To determine the bonding state of the Al-TiO_2_ nanoparticles calcined at 450 °C, X-ray photoelectron spectroscopy (XPS) measurements were performed by using a KRATOS X SAM 800 X-ray photoelectron spectrometer. Raman spectra of powdered samples were recorded at room temperature with a LabRAM HR 800 high-resolution Raman spectrometer (Horiba-Jobin Yvon) using a He-Ne laser (λ = 532 nm). The particles morphology and structure were investigated by field-emission scanning electron microscope (FE-SEM, Inspect F50, USA) with the operation voltage of 5 kV and scanning transmission electron microscopy (STEM) mode (JEOL JEM 2100 F 200 kV, equipped with a CEOS GmbH probe corrector) through high angle annual dark-field (HAADF) and bright-field (BF) imaging. The ultraviolet (UV)-visible spectra of samples were measured on a Shimadzu UV-2550 ultraviolet visible spectrophotometer.

### Photocatalytic experiments

The photocatalytic activities of the as-prepared Al-TiO_2_ nanoparticles were investigated by the degradation of acid orange 7 (AO7) in aqueous solution under irradiation of a PLS-SXE300 xenon lamp. The initial AO7 concentration was 5 mg/L with a catalyst loading of 0.5 g/L. The concentration of AO7 after photocatalytic degradation was determined by measuring the absorbance of the solution at a fixed wavelength of 484 nm, which corresponds to the maximum absorption of AO7 in the visible region. Before the absorbance measurements, the reaction solution was centrifuged for 10 min at 14000 r/min to remove the catalyst particles. The reference experiment (named as B) in the absence of catalyst and the adsorption experiment without irradiation were also performed following the procedure described above^44^.

The formation of ·OH radicals over the irradiated Al-TiO_2_ nanoparticles was examined by fluorimetry using terephthalic acid (TPA) as a ·OH radical scavenger^60^. TPA can readily react with ·OH to produce 2-hydroxyterephthalic acid (TAOH) that is a highly fluorescent compound. The photoluminescence (PL) intensity of TAOH at around 429 nm is in proportion to the amount of produced ·OH radicals. TPA was dissolved in NaOH solution (1.0 mmolL^−1^) to make a 0.25 mmolL^−1^ TPA solution. 50 mg of the catalyst was added to 100 mL of the TPA solution. After magnetically stirred for 30 min in the dark, the mixed solution was irradiated for different time. After a certain time interval, a small amount of the reaction solution was taken out and centrifuged for 10 min at 14000 rmin^−1^ to remove the catalyst. The upper clear solution in the centrifuge tube was then used for the PL measurements at a fluorescence spectrophotometer with the excitation wavelength of 315 nm.
